# A genome assembly of the speckled dace, *Rhinichthys osculus*

**DOI:** 10.1093/jhered/esag041

**Published:** 2026-05-26

**Authors:** Henry K Baker, Merly Escalona, Andrew P Kinziger, Jeff Rodzen, Steve Parmenter, Mohan P A Marimuthu, Oanh Nguyen, Noravit Chumchim, Eric Beraut, Samuel Sacco, William Seligmann, Colin W Fairbairn, Robert D Cooper, Courtney Miller, Erin Toffelmier, J Carlos Garza, H Bradley Shaffer, Jonathan B Shurin, Diana J Rennison

**Affiliations:** Department of Ecology, Behavior, and Evolution, University of California, San Diego, CA, United States; Department of Biomolecular Engineering, University of California, Santa Cruz, CA, United States; Department of Fisheries Biology, Cal Poly Humboldt, Arcata, CA, United States; Genetics Research Laboratory, California Department of Fish and Wildlife, Sacramento, CA, United States; California Department of Fish and Wildlife, Bishop, CA, United States; DNA Technologies and Expression Analysis Core Laboratory, Genome Center, University of California, Davis, CA, United States; DNA Technologies and Expression Analysis Core Laboratory, Genome Center, University of California, Davis, CA, United States; DNA Technologies and Expression Analysis Core Laboratory, Genome Center, University of California, Davis, CA, United States; Department of Ecology and Evolutionary Biology, University of California, Santa Cruz, Santa Cruz, CA, United States; Department of Ecology and Evolutionary Biology, University of California, Santa Cruz, Santa Cruz, CA, United States; Department of Ecology and Evolutionary Biology, University of California, Santa Cruz, Santa Cruz, CA, United States; Department of Ecology and Evolutionary Biology, University of California, Santa Cruz, Santa Cruz, CA, United States; Department of Ecology and Evolutionary Biology, University of California, Los Angeles, CA, United States; Department of Ecology and Evolutionary Biology, University of California, Los Angeles, CA, United States; Department of Ecology and Evolutionary Biology, University of California, Los Angeles, CA, United States; La Kretz Center for California Conservation Science, Institute for Environment and Sustainability, University of California, Los Angeles, CA, United States; Southwest Fisheries Science Center, National Marine Fisheries Service, Santa Cruz, CA, United States; Institute of Marine Sciences, University of California, Santa Cruz, CA, United States; Department of Ecology and Evolutionary Biology, University of California, Los Angeles, CA, United States; La Kretz Center for California Conservation Science, Institute for Environment and Sustainability, University of California, Los Angeles, CA, United States; Department of Ecology, Behavior, and Evolution, University of California, San Diego, CA, United States; Department of Ecology, Behavior, and Evolution, University of California, San Diego, CA, United States

**Keywords:** California conservation genomics project, ccgp, conservation genetics, cypriniformes, leuciscidae, freshwater fish

## Abstract

The speckled dace, *Rhinichthys osculus*, is a cyprinoid fish species complex (family: *Leuciscidae*) with one of the widest native ranges of any freshwater fish in western North America. It occupies a variety of freshwater habitats and exhibits considerable morphological and genetic variation across its range. Several endemic taxa within the species complex are imperiled, four of which are protected under the US Endangered Species Act, with two more proposed for listing. Here, we present an annotated, scaffold-level assembly of the speckled dace genome as part of the California Conservation Genomics Project (CCGP). Consistent with the CCGP genome assembly strategy, we used Pacific Biosciences HiFi long reads and Omni-C data for our de novo genome assembly and performed genome annotations on National Center for Biotechnology Information (NCBI) Eukaryotic Genome Annotation Pipeline using novel, species-specific RNA-Seq reads generated from five tissue types. The assembly consists of 490 scaffolds totaling approximately 1.15 Gb, with a contig N50 of 10.9 Mb and a scaffold N50 of 44.5 Mb. A BUSCO score of 97.7% reflects the assembly’s strong completeness. The genome of *R. osculus* is estimated to consist of 25 chromosomes. This is the first scaffold-level genome assembly within the genus *Rhinichthys*. We annotated a total of 39,919 genes with a BUSCO completeness score of 98.3%. This reference genome will be a valuable resource for understanding the phylogeography and evolution of speckled dace and informing its conservation.

## Introduction

The speckled dace is a minnow species complex (family: *Leuciscidae*) with one of the widest distributions of any native freshwater fish in western North America, ranging from the Colorado River basin in Sonora, Mexico, to the Columbia River Basin in southern British Columbia, Canada (Moyle 2002) (Figure 1). Speckled dace occupy a wide array of habitats, including headwater streams, large rivers, desert springs, alkaline ponds, and deep lakes. They vary morphologically across their range, often reflecting local environmental conditions (Miller 1984; Sada et al. 1995). Although genetic and morphological variation are not strongly aligned in speckled dace (Sada et al. 1995; Smith et al. 2017), they exhibit considerable genetic variation across their range (Oakey et al. 2004; Pfrender et al. 2004; Hoekzema and Sidlauskas 2014; Mussmann 2018; Su et al. 2022) and within river basins (Pfrender et al. 2004; Wiesenfeld et al. 2018). Landscape patterns of variation suggest periods of evolution in isolation punctuated by gene flow events, reflecting the complex geologic history of western North America in which basins were separated during aridification and reconnected through pluvial lake overflow, river diversions, or headwater stream capture (Smith et al. 2017). Collectively, this has produced several endemic, often cryptic lineages, some of which have been described as distinct subspecies or species, and many of which remain undescribed (Su et al. 2022; Moyle et al. 2023). Many of these endemic subspecies are critically imperiled due to habitat alteration and environmental change (Moyle 2023).

**Fig. 1:**
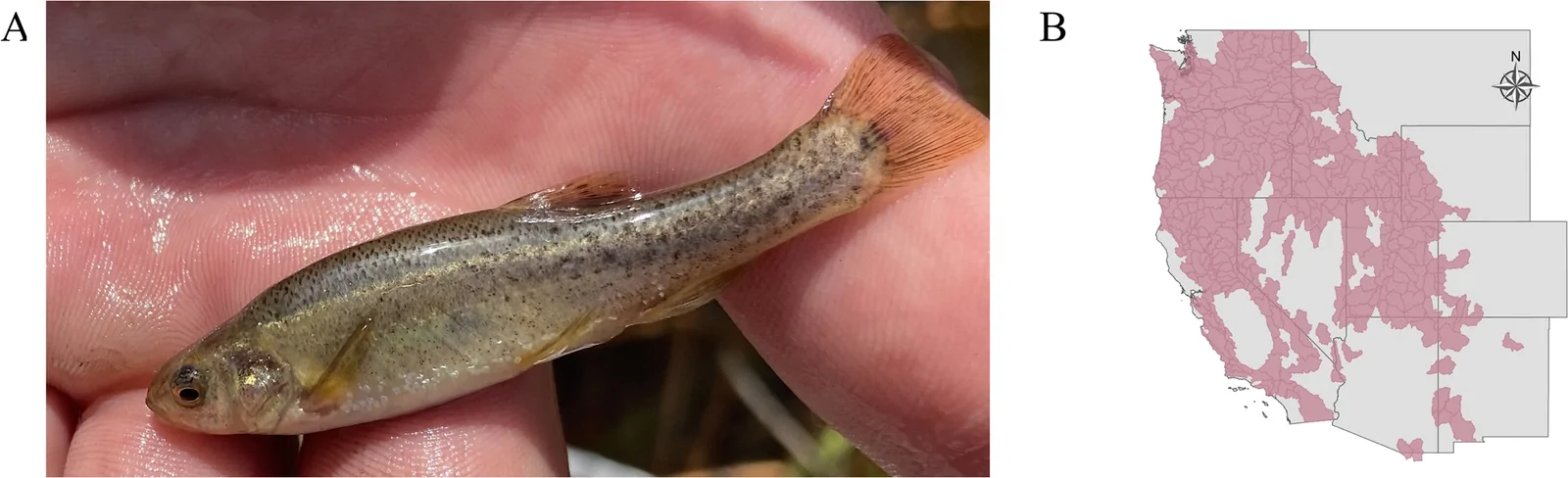
A) Photo of an adult speckled dace (*R. osculus*) from the Owens River basin near Bishop, California (Photo: H. Baker). B) Distribution of speckled dace in the United States (data from the Western Division of the American Fisheries Society; accessed via: databasin.org). Speckled dace may occur in some areas not represented in the mapped dataset, including in the mainstem Colorado River (Grand Canyon), Little Colorado River, Gila River, Salt River, Verde River basins.

Currently, four subspecies of speckled dace are listed as endangered under the US Endangered Species Act (ESA): Independence Valley speckled dace (*Rhinichthys osculus lethoporus*), Clover Valley speckled dace (*Rhinichthys osculus oligoporus*), Ash Meadows speckled dace (*Rhinichthys osculus nevadensis*), and Kendall Warm Spring speckled dace (*Rhinichthys osculus thermalis*). Additionally, in 2024 the US Fish and Wildlife Service proposed endangered status for the Long Valley speckled dace (*Rhinichthys nevadensis caldera*), which is endemic to the Long Valley Caldera near Mammoth Lakes, California, and proposed threatened status for the Santa Ana speckled dace (*Rhinichthys gabrielino*) which occupies freshwater habitats near Los Angeles, California, and was recently recognized as its own species (Moyle et al. 2023). We note that the speckled dace is a species complex currently undergoing taxonomic revision—the taxonomic names used here reflect ESA-listing documents, which in some cases are inconsistent with the most recent taxonomic revisions (Moyle 2023).

Here, we present the first annotated scaffold-level genome assembly for the speckled dace as part of the California Conservation Genomics Project (CCGP). The CCGP is a coordinated effort to build the most comprehensive conservation genomics dataset in existence, and aims to assemble reference genomes and conduct landscape genomic analyses on more than 150 of California’s native species of conservation concern (Shaffer et al. 2022). This reference genome will be a valuable resource for future studies on the biogeography, evolution, ecology, and conservation of the speckled dace.

## Methods

### Biological materials

We used one adult female fish captured in a portion of Fish Slough in the Owens River basin near Bishop, CA (37° 28′ 43.6″N 118° 24′ 09.8″W) on 10 September 2021. The fish measured 74 mm in total length and weighed 3.91 g (wet weight). The fish was transported live to the Sierra Nevada Aquatic Research Laboratory, where it was humanely euthanized using clove oil and immediately dissected on ice. Samples of several biological materials, including the caudal fin that was ultimately used for DNA extraction and sequencing, and 14 additional tissues for RNA extraction and sequencing, were dissected from the fish, flash frozen in liquid nitrogen, and stored at −80 °C. Sample aliquots were shipped on dry ice to three locations for subsequent analysis: Samples for high molecular weight (HMW) genomic DNA (gDNA) extraction and long-read sequencing were sent to the UC Davis Genome Center, samples for Omni-C sequencing were sent to UC Santa Cruz, and samples for RNA extraction and sequencing were sent to UCLA.

### Nucleic acid library preparation

HMW gDNA was extracted from the 34 mg of fin tissue using the Nanobind Tissue Big DNA kit following the manufacturer’s instructions (Pacific BioSciences [PacBio]; Menlo Park, CA). The DNA purity was estimated by absorbance ratios (260/280 = 1.90 and 260/230 = 2.42) measured using the NanoDrop ND-1000 spectrophotometer (Thermo Fisher Scientific, Waltham, MA). The DNA yield (47.2 μg) was quantified using a Quantus Fluorometer (QuantiFluor ONE dsDNA Dye assay; Promega, Madison, WI), and the size distribution of the DNA was estimated using the Femto Pulse system (Agilent, Santa Clara, CA), where 57% of the DNA fragments were found to be 30 kb or longer.

The HiFi SMRTbell library was constructed using the SMRTbell Express Template Prep Kit v2.0 (PacBio, Cat. #100-938-900) according to the manufacturer’s instructions. HMW gDNA was sheared to a target DNA size distribution between 15 and 18 kb using Diagenode’s Megaruptor 3 system (Diagenode, Belgium; cat. B06010003). The sheared gDNA was concentrated using 0.45× of AMPure PB beads (PacBio, Cat. #100-265-900) for the removal of single-strand overhangs at 37 °C for 15 min, followed by further enzymatic steps of DNA damage repair at 37 °C for 30 min, end repair and A-tailing at 20 °C for 10 min and 65 °C for 30 min, and ligation of overhang adapters v3 at 20 °C for 60 min. The SMRTbell library was purified and concentrated with 1× AMPure PB beads for nuclease treatment at 37 °C for 30 min and size selection using the PippinHT system (Sage Science, Beverly, MA; Cat #HPE7510) to collect fragments greater than 7 to 9 kb. The 15 to 20 kb average HiFi SMRTbell library was sequenced at UC Davis DNA Technologies Core (Davis, CA) using two 8 M SMRT cells, Sequel IIe sequencing chemistry 2.0, and 30-h movies each on a PacBio Sequel II sequencer.

The Omni-C library was prepared using the Dovetail Omni-C Kit (Dovetail Genomics, Scotts Valley, CA) according to the manufacturer’s protocol with slight modifications. First, specimen tissue was thoroughly ground with a mortar and pestle while cooled with liquid nitrogen. Subsequently, chromatin was fixed in place in the nucleus. The suspended chromatin solution was then passed through 100 and 40 μm cell strainers to remove large debris. Fixed chromatin was digested under various conditions of DNase I until a suitable fragment length distribution of DNA molecules was obtained. Chromatin ends were repaired and ligated to a biotinylated bridge adapter, followed by proximity ligation of adapter-containing ends. After proximity ligation, crosslinks were reversed and the DNA purified from proteins. Purified DNA was treated to remove biotin that was not internal to ligated fragments. An NGS library was generated using an NEB Ultra II DNA Library Prep kit (New England Biolabs, Ipswich, MA) with an Illumina-compatible y-adaptor. Biotin-containing fragments were then captured using streptavidin beads. The post-capture product was split into two replicates prior to PCR enrichment to preserve library complexity with each replicate receiving unique dual indices. The library was sequenced at Vincent J. Coates Genomics Sequencing Lab (Berkeley, CA) on an Illumina NovaSeq 6000 platform (Illumina, CA) to generate approximately 100 million 2 × 150 bp read pairs per gigabase of genome size.

### Genome assembly

We assembled the genome of an individual *Rhinichthys osculus* following the CCGP assembly pipeline Version 5.0. Table 1 lists the tools and non-default parameters we used in the assembly process. We removed the remnant adapter sequences from the PacBio HiFi dataset using HiFiAdapterFilt (Sim et al. 2022) and generated an initial diploid phased assembly using HiFiasm (Cheng et al. 2021, 2022) in HiC mode with the filtered PacBio HiFi reads and the Omni-C short-reads, a process that generates two assemblies, one per haplotype. We then aligned the Omni-C data to each assembly separately following the Arima Genomics Mapping Pipeline and then scaffolded each assembly with SALSA (Ghurye et al. 2017, 2019).

**Table 1:** Software used, including non-default settings.

Assembly step	Software and any non-default options	Version	Reference
**Initial assembly**			
Filtering PacBio HiFi adapters	HiFiAdapterFilt	Commit 64d1c7b	Sim et al. (2022)
K-mer counting	Meryl (*k* = 21)	1	https://github.com/marbl/meryl
Estimation of genome size and heterozygosity	GenomeScope	2	Ranallo-Benavidez et al. (2020)
De novo *assembly (contiging)*	HiFiasm (Hi-C Mode, –primary, output hic.hap1.p_ctg, hic.hap2.p_ctg)	0.16.1-r375	Cheng et al. (2021) Cheng et al. (2022)
**Scaffolding**			
Omni-C data alignment	Arima Genomics Mapping Pipeline	Commit 2e74ea4	https://github.com/ArimaGenomics/mapping_pipeline
Arima Genomics Mapping Pipeline (AGMP)	BWA-MEM	0.7.17-r1188	Li 2013
	samtools	1.11	Danecek et al. (2021)
	filter_five_end.pl (AGMP)	Commit 2e74ea4	https://github.com/ArimaGenomics/mapping_pipeline
	two_read_bam_combiner.pl (AGMP)	Commit 2e74ea4	https://github.com/ArimaGenomics/mapping_pipeline
	picard	2.27.5	https://broadinstitute.github.io/picard/
**Omni-C Contact map generation**			
Short-read alignment	BWA-MEM (-5SP)	0.7.17-r1188	Li 2013
SAM/BAM processing	samtools	1.11	Danecek et al. (2021)
SAM/BAM filtering	pairtools	0.3.0	Open2C et al. (2024)
Pairs indexing	pairix	0.3.7	Lee et al. (2022)
Matrix generation	cooler	0.8.10	Abdennur and Mirny (2020)
Matrix balancing	hicExplorer (hicCorrectmatrix correct --filterThreshold -2 4)	3.6	Ramírez et al. (2018)
Contact map visualization	HiGlass	2.1.11	Kerpedjiev et al. (2018)
	PretextMap	0.1.4	https://github.com/wtsi-hpag/PretextView
	PretextView	0.1.5	https://github.com/wtsi-hpag/PretextMap
	PretextSnapshot	0.0.3	https://github.com/wtsi-hpag/PretextSnapshot
Manual curation tools	Rapid curation pipeline (Wellcome Trust Sanger Institute, Genome Reference Informatics Team)	Commit 7acf220c	https://gitlab.com/wtsi-grit/rapid-curation
**Genome quality assessment**			
Basic assembly metrics	QUAST (--est-ref-size)	5.0.2	Gurevich et al. (2013)
Assembly completeness	BUSCO (-m geno, -l actinopterygii)	5.0.0	Manni et al. (2021)
	Merqury	29 January 2020	Rhie et al. (2020)
**Contamination screening**			
Local alignment tool	BLAST+ (-db nt, -outfmt “6 qseqid staxids bitscore std”, -max_target_seqs 1, -max_hsps 1, -evalue 1e-25)	2.15	Camacho et al. (2009)
General contamination screening	BlobToolKit (HiFi coverage, BUSCO = actinopterygii, NCBI Taxa ID = 86,924)	2.3.3	Challis et al. (2020)

The assemblies for both haplotypes were manually curated by iteratively generating and analyzing their corresponding Omni-C contact maps. Briefly, to generate the contact maps, we aligned the Omni-C data with BWA-MEM (Li 2013), identified ligation junctions, and generated Omni-C pairs (Lee et al. 2022) using pairtools (Open2C et al. 2023). Then, we generated multi-resolution Omni-C matrices with cooler (Abdennur and Mirny 2020) and balanced them with hicExplorer (Ramírez et al. 2018). We used HiGlass (Kerpedjiev et al. 2018) and the PretextSuite to visualize the contact maps. We identified misassemblies and misjoins in these contact maps, and modified the assemblies using the Rapid Curation pipeline from the Wellcome Trust Sanger Institute, Genome Reference Informatics Team. Some of the remaining gaps (joins generated during scaffolding and/or curation) were closed using the PacBio HiFi reads and YAGCloser. We checked for contamination using the BlobToolKit Framework (Challis et al. 2020).

### RNA extraction, library preparation, and sequencing

Total RNA from 14 tissues (liver, eye, pectoral fin, liver, dorsal muscle, dorsal fin, ovary, skin, heart, caudal fin, gut, fin, gill filament, and brain) was extracted using a Qiagen RNeasy Mini Kit (Qiagen, Netherlands), according to the manufacturer’s protocol. RNA libraries were then prepared using the KAPA mRNA HyperPrep Kit (Roche, Switzerland) according to the manufacturer’s protocol. Libraries were sequenced with 100 bp reads on an Illumina NovaSeq 6000 platform (Illumina, San Diego, CA), to generate approximately 50 M reads per library.

### Annotation methods

We annotated the reference assembly using the NCBI Eukaryotic Genome Annotation Pipeline v0.3.2-alpha (hereafter, “EGAPx”) which is published in the NCBI RefSeq database (O’Leary et al. 2016) and accessible through the NCBI GitHub page (“ncbi/egapx”). Annotation features were identified by aligning transcripts and proteins from related taxa in the RefSeq database using BLAST (Camacho et al. 2009). Novel, species-specific RNA-Seq reads generated from five tissue types were also aligned to the assembly using the alignment software STAR (Dobin et al. 2013). Additional features were predicted using HMM-based gene models using the NCBI Gnomon software. We evaluated the quality and completeness of our annotation by comparing the longest protein for each annotated coding gene to eukaryotes (odb12) and actinopterygii (odb12) using BUSCO v5.8.2 (Manni et al. 2021). We report the number of annotation features and the BUSCO results in Table 2.

**Table 2:** Sequencing and assembly statistics, and accession numbers.

Bio Projects & Vouchers	CCGP NCBI BioProject			PRJNA720569			
	Generus NCBI BioProject			PRJNA765855			
	Species NCBI BioProject			PRJNA808373			
	NCBI genome BioSample			SAMN34081523			
	NCBI RNA BioSamples			SAMN40863748-61			
	Specimen identification			CCGP_106_AS_Ref1			
	NCBI genome accessions			**Haplotype 1**		**Haplotype 2**	
	Assembly accession			JARWKJ000000000		JARWKK000000000	
	Genome sequences			GCA_029959095.1		GCA_029959135.1	
Genome sequence	PacBio HiFi reads	Run		1 PACBIO_SMRT (Sequel II) run: 4.5M spots, 62.2G bases, 37.7G bytes			
		Accession					SRX21227379
	Omni-C Illumina reads	Run		2 ILLUMINA (Illumina NovaSeq 6000) runs: 104.1M spots, 31.5G bases, 10G bytes			
		Accession					SRX21227380-1
Genome assembly quality Metrics	Assembly identifier (Quality code^a^)				fRhiOsc1(7.7.P7.Q64.C98)		
	HiFi Read coverage^b^						56.85×
				**Haplotype 1**		**Haplotype 2**	
	Number of contigs			737		459	
	Contig N50 (bp)			10,885,064		10,040,549	
	Contig NG50^b^			11,607,600		10,415,998	
	Longest Contigs			34,281,686		34,966,866	
	Number of scaffolds			490		187	
	Scaffold N50			44,510,230		43,302,895	
	Scaffold NG50^b^			44,510,230		43,302,895	
	Largest scaffold			68,221,045		66,459,167	
	Size of final assembly			1,145,805,923		1,144,992,768	
	Phased block NG50^b^			11,195,889		10,588,430	
	Gaps per Gbp (# Gaps)			216 (247)		238 (272)	
	Indel QV (Frame shift)			49.52642159		50.9193815	
	Base pair QV			64.0805		64.7274	
				Full assembly = 64.3918			
	k-mer completeness			86.7458		86.7235	
				Full assembly = 98.9575			
	BUSCO completeness^c^ (actinopterygii) *n* = 3640		**C**	**S**	**D**	**F**	**M**
		H1^d^	97.70%	96.40%	1.30%	0.90%	1.40%
		H2^d^	97.70%	96.40%	1.30%	0.90%	1.40%
Genome annotation quality metrics				**Count of features**			
	Genes				39,919		
	Transcripts				59,926		
	mRNA				44,064		
	lncRNA				15,606		
	CDSs				44,074		
	BUSCO completeness^c^		**C**	**S**	**D**	**F**	**M**
	eukaryota_odb12		99.2%	98.4%	0.8%	0.0%	0.8%
	actinopterygii_odb12		98.3%	97.2%	1.1%	0.7%	1.0%

^a^Assembly quality code x.y.P.Q.C derived notation, from (Rhie et al. 2021). *x* = log10[contig NG50]; *y* = log10[scaffold NG50]; *P* = log10 [phased block NG50]; *Q* = Phred base accuracy QV (Quality value); *C* = % genome represented by the first “n” scaffolds, following a known karyotype for this species of 2*n* = 50 (Genome on a Tree, query (*Rhinichthys osculus*); Challis et al. 2023). Quality code for all the assembly denoted by primary assembly (bRhiOsc1.0.hap1)

^b^Read coverage and NGx statistics have been calculated based on the estimated genome size of 1.09 Gb

^c^BUSCO Scores. Complete BUSCOs (C). Complete and single-copy BUSCOs (S). Complete and duplicated BUSCOs (D). Fragmented BUSCOs (F). Missing BUSCOs (M).

^d^(H1) Haplotype 1 and (H2) Haplotype 2 assembly values.

Additional details on the CCGP genome assembly pipeline can be found at (https://www.ccgproject.org/genome-assembly-pipeline).

## Results

### Sequencing data

The Omni-C library generated 104.16 million read pairs, and the PacBio HiFi library generated 4.54 million reads. The PacBio HiFi sequences yielded ~56× genome coverage and had an N50 read length of 14,211 bp, a minimum read length of 149 bp, a mean read length of 13,673 bp, and a maximum read length of 57,885 bp (see read length distribution in Supplementary Fig. S1). Based on the PacBio HiFi data, Genomescope 2.0 estimated a genome size of 1.09 Gb, a 0.38% sequencing error rate, and 0.973% heterozygosity. The k-mer spectrum shows a bimodal distribution with a major peak at ~55-fold coverage and a minor peak at ~27-fold coverage (Fig. 2A). Sequencing yields of mRNA libraries for each of the 14 samples are shown in Supplementary Table S1.

**Fig. 2:**
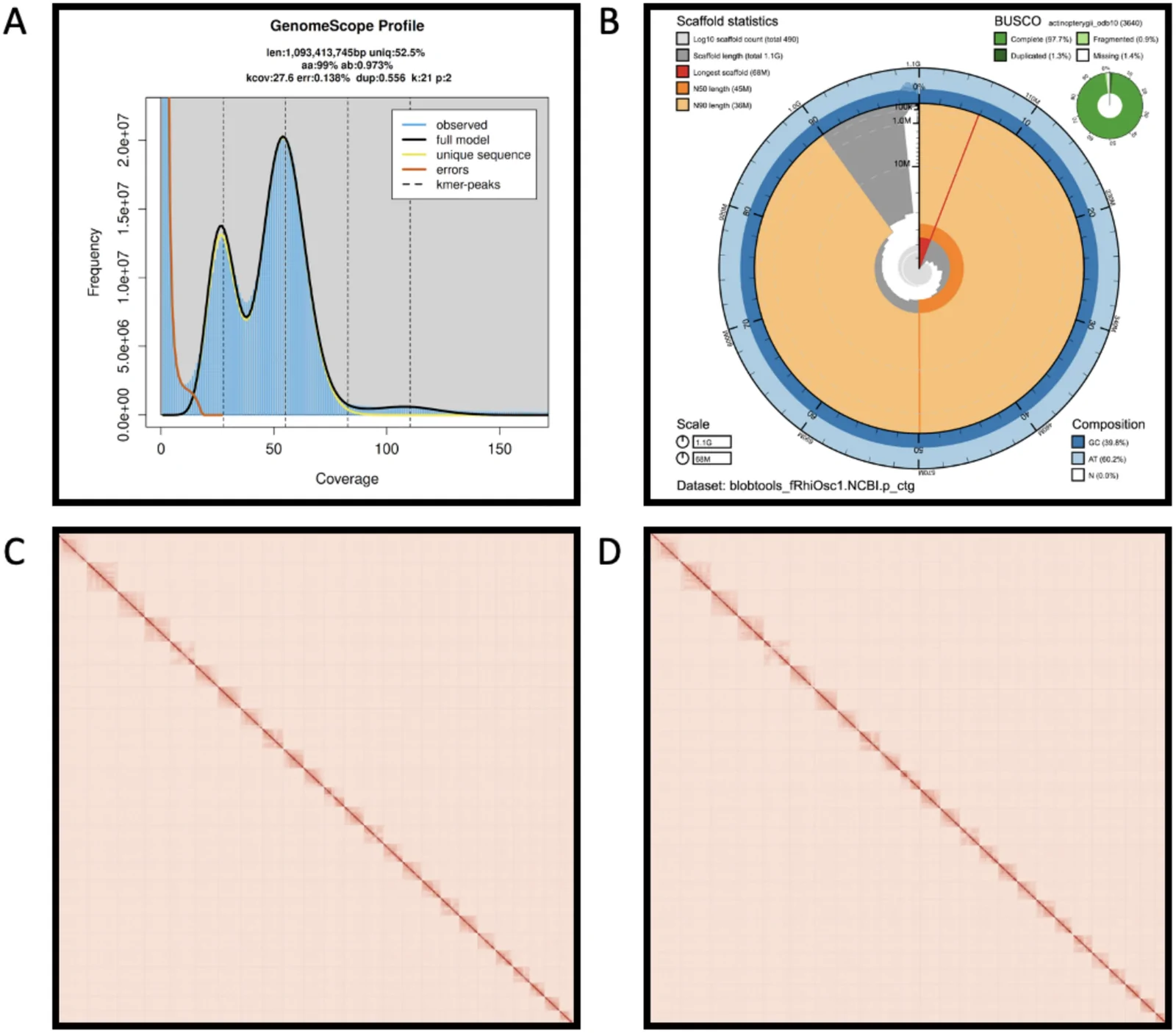
Visual overview of genome assembly metrics for the *R. osculus* primary assembly (fRhiOsc1). A) Kmer spectrum output generated from PacBio HiFi data without adapters using GenomeScope 2.0. The observed distribution is characteristic of an outbred diploid genome, with bimodality indicating the presence of heterozygosity. B): BlobToolKit Snailplot showing a graphical representation of the quality metrics outlined in Table 2 for the primary *R. osculus* assembly and BUSCO scores for the set of orthologues. The plot circle indicates the full size of the assembly. Moving from the center outwards, the central plot depicts length-related metrics. The red line denotes the size of the longest scaffold. All other scaffolds are arranged in size order, moving clockwise around the plot, and drawn in gray starting from the outside of the central plot. The dark and light orange arcs highlight the scaffold N50 and scaffold N90 values. The central light gray spiral indicates the cumulative scaffold count with a white line at each order of magnitude. White regions in this area reflect the proportion of Ns in the assembly; the dark versus light blue area around it depicts the mean, maximum, and minimum GC vs. AT content at 0.1% intervals (Challis et al. 2020). Omni-C contact maps for the primary C) and alternative D) genome assembly generated using PretextSnapshot. These contact maps translate proximity of genomic regions in 3D space to contiguous linear organization. Each cell in the contact map corresponds to sequencing data supporting the linkage between two of such regions.

### Nuclear genome assembly

The final genome assembly (fRhiOsc1) consists of two phased haplotypes; both assemblies are similar in size, and both are similar but not equal to the estimated genome size from GenomeScope2.0, as has been observed in other taxa (see Pflug et al. 2020, for example).

The haplotype one assembly (fRhiOsc1.0.hap1) consists of 490 scaffolds spanning 1.15 Gb with a contig N50 of 10.89 Mb, a scaffold N50 of 44.51 Mb, the largest contig size of 34.28 Mb, and the largest scaffold size of 68.22 Mb. The haplotype two assembly (fRhiOsc1.0.hap2) consists of 187 scaffolds spanning 1.14 Gb with a contig N50 of 10.04 Mb, a scaffold N50 of 43.30 Mb, the largest contig size of 34.97 Mb and the largest scaffold size of 66.46 Mb.

The haplotype one assembly has a BUSCO completeness score for the *Actinopterygii* gene set of 97.7%, a base pair quality value (QV) of 64.08, a k-mer completeness of 86.75%, and a frameshift indel QV of 49.53. The haplotype two assembly has a BUSCO completeness score for the *Actinopterygii* gene set of 97.7%, a base pair QV of 64.72, a k-mer completeness of 86.72%, and a frameshift indel QV of 50.91.

During manual curation, we made a total of 223 joins (109 on haplotype one and 114 on haplotype two) and 27 breaks (13 on haplotype one and 14 on haplotype two) based on the Omni-C contact map signal. We closed a total of 72 gaps (32 on haplotype one and 40 on haplotype two), and we filtered out 2 contigs corresponding to contamination from *Pimephales* minnow adintovirus virus (cumulative size: 680,560 bp; matched to NCBI: BK011009.1). No other contigs were removed or modified.

The Omni-C contact maps show highly contiguous assemblies, with chromosome-length scaffolds, suggesting that the genome of the *R. osculus* is organized in 25 chromosomes based on the number of major bins along the diagonal axis of the contact maps (Fig. 2B). Assembly statistics are reported in Table 2 and represented graphically in Fig. 2C. We have deposited the genome assembly on NCBI GenBank (see Table 2 and Data availability for details).

Our final genome annotation included 39,919 genes, with an actinopterygii BUSCO completeness of 98.3%. A list of annotation statistics and BUSCO score breakdowns for eukaryotes and actinopterygii is reported in Table 2.

## Discussion

The genome assembly for the speckled dace (*R. osculus*) is an important contribution to the genomic characterization of western North American freshwater fishes. As one of the most widely distributed native minnows in the western United States, the speckled dace occupies an extraordinary array of ecological settings, from cold mountain streams to isolated desert springs (Moyle 2002). This ecological breadth has led to considerable morphological and genetic diversity across its range (Miller 1984; Sada et al. 1995; Oakey et al. 2004; Smith et al. 2017), making *R. osculus* an interesting system in which to study adaptation, speciation, and freshwater conservation. Although recent studies using de novo assemblies from RADseq data have improved our understanding of the phylogeography of speckled dace (e.g. Mussmann et al. 2020; Su et al. 2022), the availability of an annotated genome assembly will facilitate research investigating the genetic basis of ecologically relevant traits such as thermal tolerance and adaptations to slow- versus swift-water habitats.

From a conservation perspective, this genome is especially timely. Many isolated populations of speckled dace, particularly those inhabiting desert springs and terminal basins, are highly vulnerable to habitat loss, groundwater depletion, and climate change. Some of these populations are currently being considered as candidates for listing under the ESA due to their unique evolutionary histories and limited geographic distributions. With a reference genome in hand, conservationists and researchers can more readily assess genomic diversity, inbreeding, and adaptive differentiation with far greater resolution. This enables more accurate delineation of distinct population segments, which are key to making sound, and defensible conservation decisions. Genome-wide markers have already been useful for elucidating cryptic ESUs in California speckled dace (Mussmann et al. 2020, Su et al. 2022; Moyle et al. 2023); using restriction site associated DNA sequencing (RADseq) researchers identified the Santa Ana speckled dace and Long Valley speckled dace lineages, which have recently been proposed for ESA listing. Additional whole genome data will help to further resolve taxonomic uncertainty and help to delineate which units of variation warrant conservation.

Currently, there are 41 other publicly available reference genomes for species within *Leuciscidae*, a diverse family comprising minnows from North America, Europe, and Asia, including nine from the subfamily *Pogonichthyinae,* which contains *Rhinichthys*. One other reference genome exists within the genus *Rhinichthys,* which is of relatively high quality (BUSCO completeness = 94.7%), but contig-level assembly (*Rhinichthys klamathensis goyatoka*; unpublished). The *R. osculus* scaffold-level assembly, with a BUSCO completeness score of 97.7%, enhances the available genomic resources for the genus and supports more detailed comparative and population-level analyses. The *R. osculus* genome size estimated from our assembly was 1.15 Gb, which is similar to other genome assemblies within *Leuciscidae* (mean = 1.02 Gb, range: 0.62 to 1.32 Gb). The estimated diploid chromosome number in *R. osculus* aligns with the standard count of 2*n* = 50 reported for most North American cyprinids (Amemiya et al. 1992). The estimated gene count of 39,919 appears somewhat high relative to the estimated genome size. We note that some automated annotation pipelines have been associated with overprediction of gene counts, but those issues are mitigated with more complete annotations and higher quality data, including RNA-seq data as we used here. Gene count estimates should be interpreted in light of the annotation quality metrics such as BUSCO completeness. Our genome annotation BUSCO score of 98.3% is consistent with a high-quality annotation.

This reference genome enhances our capacity for phylogenomic and biogeographic studies across the western United States, a region with high endemism and rapid speciation in freshwater fishes. As more reference genomes become available, this speckled dace genome serves as an anchor for investigating patterns of parallel evolution, genomic responses to isolation, and hybridization dynamics across the landscape. Additionally, because the genome is annotated it will serve as a useful tool for comparative genome studies. Ultimately, this resource will strengthen both evolutionary research and our ability to conserve freshwater biodiversity in the face of accelerating environmental change.

## Supplementary Material

Supplementary_material_esag041

## Data Availability

Data generated for this study are available under NCBI BioProject PRJNA808373. Raw sequencing data for sample CCGP_106_AS_Ref1 (NCBI BioSample SAMN34081523) are deposited in the NCBI Short Read Archive (SRA) under SRR25496294 for PacBio HiFi sequencing data, and SRR25496292-93 for the Omni-C Illumina sequencing data. GenBank accessions for both primary and alternate assemblies are GCA_029959095.1 and GCA_029959135.1; and for genome sequences JARWKJ000000000 and JARWKK000000000. Assembly scripts and other data for the analyses presented can be found at the following GitHub repository: www.github.com/ccgproject/ccgp_assembly. Data generated for the annotation in this study are available under NCBI BioProject PRJNA1011957. Raw RNA sequencing data (NCBI BioSamples SAMN40863761, SAMN40863752, SAMN40863759, SAMN40863757, SAMN40863751, SAMN40863750, SAMN40863758, SAMN40863760, SAMN40863756, SAMN40863749, SAMN40863755, SAMN40863753, SAMN40863754, SAMN40863748) are deposited in the NCBI SRA [SRR29116064, SRR29116061, SRR29116066, SRR29116056, SRR29116062, SRR29116063, SRR29116067, SRR29116065, SRR29116057, SRR29116068, SRR29116058, SRR29116060, SRR29116059, SRR29116069], respectively).
