# Supplementary material for: A genome assembly of the speckled dace, Rhinichthys osculus
Source: J Hered. 2026 May 26;117(6):1316–24. doi: 10.1093/jhered/esag041 (PMC13733543; doi:10.1093/jhered/esag041)
Supplement: Supplementary file 1 [file supplementary_material_esag041.zip › Supplemental_Figure_esag041.docx]

***Supplemental Figures:***

Figure S1. Read length distribution for speckled dace (*R. osculus*) from PacBio genome sequencing.
