# Supplementary material for: A genome assembly of the speckled dace, Rhinichthys osculus
Source: J Hered. 2026 May 26;117(6):1316–24. doi: 10.1093/jhered/esag041 (PMC13733543; doi:10.1093/jhered/esag041)
Supplement: Supplementary file 1 [file supplementary_material_esag041.zip › Supplemental_Table_esag041.docx]

# ***Supplemental Tables***

Table S1. mRNA sequencing yields by sample.

| Sample ID | Paired-end reads (millions) |
| --- | --- |
| SAMN40863761 | 48 |
| SAMN40863752 | 50 |
| SAMN40863759 | 46 |
| SAMN40863757 | 51 |
| SAMN40863751 | 46 |
| SAMN40863750 | 81 |
| SAMN40863758 | 60 |
| SAMN40863760 | 46 |
| SAMN40863756 | 47 |
| SAMN40863749 | 45 |
| SAMN40863755 | 97 |
| SAMN40863753 | 47 |
| SAMN40863754 | 44 |
| SAMN40863748 | 54 |
